# Successes and challenges in clinical gene therapy

**DOI:** 10.1038/s41434-023-00390-5

**Published:** 2023-11-08

**Authors:** Donald B. Kohn, Yvonne Y. Chen, Melissa J. Spencer

**Affiliations:** 1 Department of Microbiology, Immunology & Molecular Genetics, David Geffen School of Medicine, University of California, Los Angeles, Los Angeles, CA USA; 2 Department of Pediatrics, David Geffen School of Medicine, University of California, Los Angeles, Los Angeles, CA USA; 3 The Eli and Edythe Broad Center for Regenerative Medicine and Stem Cell Research, University of California, Los Angeles, Los Angeles, CA USA; 4 Department of Chemical and Biomolecular Engineering, Henry Samueli School of Engineering, University of California, Los Angeles, Los Angeles, CA USA; 5 Parker Institute for Cancer Immunotherapy Center at UCLA, University of California, Los Angeles, Los Angeles, CA USA; 6 Department of Neurology, David Geffen School of Medicine, University of California, Los Angeles, Los Angeles, CA USA

**Keywords:** Tumour immunology, Haematological diseases, Neurological disorders

## Abstract

Despite the ups and downs in the field over three decades, the science of gene therapy has continued to advance and provide enduring treatments for increasing number of diseases. There are active clinical trials approaching a variety of inherited and acquired disorders of different organ systems. Approaches include ex vivo modification of hematologic stem cells (HSC), T lymphocytes and other immune cells, as well as in vivo delivery of genes or gene editing reagents to the relevant target cells by either local or systemic administration. In this article, we highlight success and ongoing challenges in three areas of high activity in gene therapy: inherited blood cell diseases by targeting hematopoietic stem cells, malignant disorders using immune effector cells genetically modified with chimeric antigen receptors, and ophthalmologic, neurologic, and coagulation disorders using in vivo administration of adeno-associated virus (AAV) vectors. In recent years, there have been true cures for many of these diseases, with sustained clinical benefit that exceed those from other medical approaches. Each of these treatments faces ongoing challenges, namely their high one-time costs and the complexity of manufacturing the therapeutic agents, which are biological viruses and cell products, at pharmacologic standards of quality and consistency. New models of reimbursement are needed to make these innovative treatments widely available to patients in need.

## Successes and challenges in gene therapy—inherited blood cell disorders

### Hematopoietic stem cell transplantation (HSCT) for inherited blood cell diseases

Inherited blood cell diseases were the first group of disorders approached and successfully treated with gene therapy. These monogenic diseases affect the production of blood cells or their function and include 1) hemoglobinopathies that affect red blood cells (sickle cell disease, thalassemia); 2) inborn errors of immunity (IEI) affecting neutrophils, macrophages or lymphocytes; 3) lysosomal storage diseases and some leukodystrophies affecting tissue resident macrophages and brain microglial cells, and 4) conditions that lead to impaired HSC function and genome stability (Fanconi Anemia).

These inherited blood cell diseases can be cured by transplanting normal hematopoietic stem cells (HSC) from a suitably matched healthy (allogeneic) donor that can engraft and produce normal blood cells. The donor HSC are infused into the bloodstream of the patient and will home to the bone marrow space, where they take up life-long residence and produce the needed blood cells that were deficient. Performing HSC transplantation for blood cell diseases requires the availability of a suitably matched donor to minimize risks of unwanted immunological reactions between the immune cells of the donor and the recipient, such as graft rejection or graft versus host disease. The outcomes of HSC transplants have progressively improved over the last several decades, due to improved methods for tissue typing, better methods for conditioning the recipient with chemotherapy to “make space” for the engraftment of the donor HSC in the bone marrow, and improved supportive measures, such as antibiotics, antifungal and antiviral drugs, and nutritional support. However, lack of suitable matched donors and the immunological complications limit outcomes for allogeneic HSC transplantation.

### Hematopoietic stem cell gene therapy (HSCGT)

Hematopoietic stem cell gene therapy (*HSCGT*) for inherited blood disorders uses the patient’s own (autologous) HSC that are gene corrected either by adding a normal copy of the inherited defective gene with an integrating vector or, more recently, editing the defective gene to restore its function. Because HSC can be removed from the body by bone marrow harvest or blood stem cell collection, gene modified ex vivo, and then returned to the patient by intravenous infusion, relatively high levels of engraftment of gene-corrected HSC can be achieved (25->90%). HSCGT has shown clinical efficacy for a growing list of disorders (Table 1) [1, 2] and there are many additional related disorders with gene-specific therapies for them under early clinical or pre-clinical development. HSCGT for X-linked adrenoleukodystrophy (Skysona™) and β-thalassemia (Zynteglo™), recently received U.S. FDA marketing approval.

**Table 1:** Trials with positive clinical benefits with HSCGT.

Disorder	References
1. X-Adrenoleukodystrophy	[18]
2. Metachromatic Leukodystrophy	[19]
3. X-linked SCID	[10, 11]
4. Adenosine Deaminase SCID	[12]
5. Artemis SCID	[13]
6. Wiskott-Aldrich Syndrome	[14, 15]
7. X-linked Chronic Granulomatous Disease	[16]
8. Leukocyte Adhesion Deficiency I	[17]
9. Beta-Thalassemia	[21, 22]
10. Sickle Cell Disease	[25–28]
11. MPS-I (Hurler’s Syndrome)	[20]
12. MPS IIIb (Sanfilippo Syndrome)	[42]
13. Fabry’s Disease	[43]
14. Pyruvate Kinase Deficiency	[44]
15. Fanconi Anemia A	[36]

### Successes

*Severe combined immune deficiency (SCID)* was the first clinical success with gene therapy. SCID is the most severe human IEI, with absent T and B lymphocyte function making the infant susceptible to life-threatening infections with high mortality in the absence of treatment. It is the first genetic blood cell disease to be curatively treated with allogeneic HSC transplantation, with bone marrow transplants from matched siblings being highly effective at restoring immunity [3, 4]. But the majority of SCID patients do not have a matched sibling donor and are treated with transplants from alternative donors including haplo-identical parents, or well-matched unrelated donors. Survival with sustained immunity after these types of transplants has been lower than with sibling donors, but outcomes are continually improving [4, 5]. Nevertheless, autologous gene therapy may provide an effective and safe treatment.

Initial successes to restore immunity were achieved using murine gamma-retrovirus vectors to transfer the genes for XSCID (*IL2RG*) and ADA SCID (*ADA*) into patient’s bone marrow HSC, with clinically beneficial immune reconstitution and good health [6, 7]. However, two or more years after gene therapy, 6 of 20 XSCID patients developed a serious complication of leukemia induced by the vector [8, 9], and once recently for ADA SCID [1].

The field shifted to the use of lentiviral vectors for their lower risks for genotoxicity compared to gamma-retroviral vectors, as well as more effectiveness for transducing human HSC. In fact, the recent clinical results with gene therapy for XSCID, ADA SCID as well as Artemis SCID (*DCLRE1C*) using lentiviral vectors have been excellent [10–13]. Across multiple studies in France, the U.K. and the U.S. there are consistently very high frequencies of survival with successful immune reconstitution. There is also lower transplant acuity with gene therapy compared to alternative donor transplants because reduced intensity conditioning is used, without immune suppression. Other IEIs have been successfully treated with HSCGT using lentiviral vectors, including Wiskott-Aldrich syndrome, chronic granulomatous disease, and leukocyte adhesion deficiency I [14–17].

Excellent clinical results have also been achieved for several metabolic disorders (lysosomal storage and leukodystrophies) in which monocyte-derived cells—macrophages, microglia—are involved in disease pathogenesis [18–20]. In several of these disorders, (e.g., metachromatic leukodystrophy, mucopolysaccharidosis I) the transgene leads to over-expression of the gene product which can be released from the transduced blood cells and cross-correct other somatic cells. This over-expression and high-level cross-correction do not occur using allogeneic healthy donors, and thus HSCGT may produce superior results for these diseases, as has been indicated for metachromatic leukodystrophy [20].

The hemoglobinopathies, sickle cell disease and β- and α-thalassemia, are important disease targets for gene therapy as these disorders are more common than the IEI and metabolic disorders. For β-thalassemia, lentiviral vectors have been developed expressing β-globin genes that supplement the deficient endogenous β-globin production. The published trials of one lentiviral vector for β-thalassemia showed high rates of improvement in red blood cell production, to allow transfusion therapy to be stopped for most treated patients [21, 22]. This is the treatment for β-thalassemia HSGCT (Zynteglo™) that was recently approved by the U.S. FDA, mentioned above.

Most gene therapy approaches for sickle cell disease are based on the clinical observation that expression of increased amounts of fetal hemoglobin moderate the severity of sickle cell disease, attributed to the ability of fetal hemoglobin to slow the rate of aggregation of deoxyhemoglobin S [23, 24]. The γ-globin chain of fetal hemoglobin has a specific amino acid (Q87) that is responsible for interference with HbS aggregation. Lentiviral vectors expressing “anti-sickling genes” (γ-globin transgene or β-globin substituted with the Q87 amino acid from γ-globin) that impede aggregation of sickle hemoglobin have been shown to have clinical benefits, with significant reduction in acute complications of sickle cell disease [25, 26]. Other approaches used a short hairpin RNA to reduce the mRNA for the repressor of γ-globin expression, *BCL11a*, inducing high levels of fetal globin production and greatly reducing acute sickle complications [27]. CRISPR/Cas9 is being used to disrupt the erythroid enhancer element for the *BCL11a* gene to induce increased γ-globin production and to facilitate correction of the sickle cell-causing mutation in the *HBB*^S^ gene by homology-directed repair [28–30]. Base-editing trials advancing to the clinic will be used to either eliminate the BCL11a-binding sites at the γ-globin genes and thereby induce fetal globin; or to convert the codon containing the sickle cell-causing mutation to one encoding an amino acid that does not cause sickling [31, 32].

#### Opportunities

There are many more inherited blood cell diseases for which lentiviral vector gene therapies are being developed, including additional IEI, α-thalassemia, storage and metabolic disorders. Gene editing technologies are advancing at break-neck speed, with nuclease-mediated editing (zing finger nucleases, TALENS, CRISPR/Cas9) being followed by base editing, prime editing and more to come. The ability to make precise edits in endogenous genes should confer more physiological expression of genes needed for safe and effective HSCGT.

### Challenges

#### Safety risks

In terms of safety issues, a hypothetical concern about lentiviral vectors recombining with the lentiviral genes used for packaging to produce a replication-competent lentivirus capable of spreading infection has never been reported [33].

A major safety concern for HSCGT is the risks of genotoxicity from the integrating vector. In fact, early trials using gamma-retroviral vectors for XSCID, CGD, and WAS had serious complications from the vector causing leukemia in some of the patients [1, 2]. Lentiviral vectors have been markedly safer across multiple clinical trials for more than a dozen disorders. In general, integration site analyses do not show preferential integration near oncogenes, nor clinically significant clonal expansion. The only clinically significant genotoxicities with lentiviral vectors have occurred with vectors that contained the long terminal repeat enhancer elements from gamma-retroviruses, which are the element responsible for their genotoxicity; or with inclusion of an insulator element that inadvertently acts as a splice acceptor-polyA signal and can cause premature truncation of cellular transcripts when the vector integrates into introns [34, 35]. Vectors of many other designs, using promoters of cellular housekeeping genes with low enhancer activity (e.g. human phosphoglycerate kinase, or elongation factor-1α) or with lineage-restricted expression (e.g. beta-globin, chimeric myeloid) have not shown genotoxicity, but yield a polyclonal vector distribution without clonal expansions [1, 2].

It is postulated that use of site-specific gene editing may have significantly reduced risks of genotoxicity compared to randomly integrated lentiviral vectors. However, gene editing methods also have inherent genotoxicity risks, including disruptive insertions and deletions of various sizes at editing sites, loss of chromosomal material distal to a nuclease cleavage site, bystander edits in the region targeted by base editors, and off-target editing. The risks for any specific editing strategy need to be considered and assessed as part of clinical translation of gene editing for a clinical cell product.

#### Complexity

The manufacture of gene-modified autologous HSC drug products is complex. A patient-specific stem cell collection is needed for each autologous product. These procedures have moderate clinical complexity, entailing 5–7 days of receiving G-CSF for mobilization, placement of a suitable central venous pheresis catheter, and 1–3 leukapheresis sessions.

CD34 + cell selection and either lentiviral vector transduction or gene editing with reagents introduced by electroporation are relatively standardized, but entail many hours of cell processing in the clean room facility. There is a relatively low rate of lot failure, with criteria for purity, potency, identity and safety met in most cases. And, administration of the gene-modified stem cells in the context of a clinical stem cell transplantation with moderate to intense conditioning chemotherapy requires high acuity in-patient medical care for several weeks. However, the clinical standard of care approach for these disorders is an allogeneic HSCT which has similar (or greater) clinical complexity.

#### Conditioning

Conditioning chemotherapy is routinely used as pre-transplant in nearly all blood-cell diseases to attain sufficient engraftment of the gene-modified HSCs, with Fanconi anemia being an exception that can achieve therapeutic levels of stem-cell engraftment without conditioning due to the stem-cell defects inherent in the disease [36]. The conditioning imposes risks of organ toxicity, infections, pancytopenia requiring transfusions and often antibiotics, as well as discomforts including mucositis, nausea, vomiting, anorexia and alopecia. While conditioning chemotherapy also carries risks from potential mutagenic effects of the alkylating agents, at least one report of a cohort of ADA SCID patients who received reduced-intensity conditioning with busulfan did not display any mutations in the blood cells typical of clonal hematopoiesis [37]. Nevertheless, there is a great deal of effort to replace chemotherapy with less toxic conditioning agents, such as monoclonal antibodies to stem cell markers (e.g., CD117, CD45), either unconjugated or as antibody-drug conjugates [38–40]. Effective conditioning without chemotherapy should significantly improve the safety profile of these autologous transplants.

#### Variability

There is significant variability in the levels of engraftment of gene-corrected cells across members in different study cohorts in reported gene therapy trials [14, 15, 19, 26]. We have shown that, in an ADA trial, the level of engraftment of gene-modified stem cells (based on vector copy number in granulocytes) was a function of the CD34 + cell dose, the percentage of the CD34 + cells that were transduced, and the intensity of the conditioning, based on the area-under-the-curve for busulfan exposure [41]. Thus it is important to optimize each component of the gene therapy procedure for optimal results.

#### Cost

HSCGT is delivered in the context of autologous HSC transplant, and the costs for the clinical component are relatively standard, including screening, clinical labs, central venous line placement, administration of conditioning chemotherapy, the post-transplant clinical care including the costs for in-patient stay on ICU-like transplant units plus costs for antibiotics, parental nutrition if needed, lab and infectious disease testing, radiology studies, etc. The unique components of cost are those for manufacturing the cell product, including the vector lot, the cell processing costs (materials for CD34 selection, cell culture, testing, GMP facility, regulatory oversight). In the academic setting many of these costs are much lower than in the commercial setting where the expectations for the quality of the documentation, facility building costs, maintenance and oversight, and a much higher level of staffing raise the costs.

#### Commercialization

The major challenges to developing and employing these gene therapies are not technical but financial. The costs are high for reagents like clinical-grade lentiviral vectors or gene editing reagents, as well as for the cell processing materials and Good Manufacturing Practices (GMP) facility and personnel costs, in addition to the drug research and development costs. It is anticipated that costs per patient dose may be reduced as the methods for vector production and cell processing are improved. The improved safety and reduction in clinical costs and improved outcomes using these autologous gene-corrected HSC products need to exceed those from the various allogeneic HSCT options that are available. Certainly, avoiding the use of potent immune suppressive drugs pre- and post-transplant and the absence of risks for graft versus host disease may provide this competitive edge to gene therapies, but this is to be determined.

## Successes and challenges in gene therapy—chimeric antigen receptor T cells (CARs)

### Redirecting immune cells against cancer

Immunotherapy, or treatment that engages or redirects the immune system against diseased cells, has become a fourth pillar in cancer immunotherapy alongside chemotherapy, radiation, and surgery. In addition to small-molecule drugs that interface with immune-cell function [42] and protein biologics such as cytokines and checkpoint-blockade antibodies [43, 44] cell-based immunotherapy has emerged as a potent new tool in the immuno-oncology arsenal. Here, we provide a brief overview of the successes and challenges of genetically modified cell-based therapy for cancer.

Cell-based immunotherapy can be conceptualized as the engineering of a chassis (i.e., the immune cell) to execute anti-tumor operations with the assistance of programmed software (i.e., transgenic elements that encode specified functions). The development of antigen-specific receptors that redirect engineered immune cells against tumor cells forms the foundation of cell-based cancer immunotherapy. Various immune cell types—including T cells, natural-killer (NK) cells, and macrophages—have been engineered to express tumor-targeting receptors, expanded ex vivo, and infused into cancer patients to achieve targeted tumor eradication[45–47]. To date, T cells serve as the most commonly used chassis in cell-based immunotherapy, and two main categories of receptors have been used to redirect T-cell specificity toward cancer: T-cell receptors (*TCRs*) and chimeric antigen receptors (*CARs*).

TCRs are naturally occurring receptors that define the antigen specificity of T cells. Pioneering work in T-cell therapy led to the discovery that tumor-infiltrating lymphocytes (TILs), which express endogenous TCRs recognizing tumor-specific or tumor-associated antigens, can be isolated from cancer patients, expanded ex vivo, and re-infused into the same patient to augment the antitumor response [48, 49]. Extending beyond TIL isolation and expansion, one could identify tumor-reactive clones among TILs, isolate the tumor-reactive TCR sequence, and introduce a transgenic copy of the TCR gene into non–tumor-specific T cells to artificially confer tumor-recognition capability [50, 51]. Furthermore, a suite of technologies has been developed to identify and isolate tumor-reactive TCRs via high-throughput in vitro library screening. For example, multiplexed single-cell transcriptomics and TCR sequencing yielded the identification of TCR clones that recognize a “public” neoantigen derived from *PIK3CA*, with clinical applicability to patients with diverse malignancies ranging from uterine serous carcinoma to colon adenocarcinoma to anaplastic thyroid cancer [52]. This approach begins with a known target antigen, and search for cognate TCRs. The reverse process—i.e., starting with a tumor-reactive TCR in search of a cognate ligand—can also be accomplished through the use of peptide-major histocompatibility complex (MHC) libraries presented by target cells [53].

Unlike TCRs, CARs are synthetic proteins comprising heterologous parts, and they can recognize antigens in an MHC-independent manner [54]. Importantly, CARs have been shown to function not only in T cells, but also in NK cells [55], macrophages [56], and neutrophils [57]. The antigen-specificity of CAR molecules is dictated by its extracellular ligand-binding domain, which is most commonly a single-chain variable fragment derived from a monoclonal antibody that binds the antigen of interest. The ligand-binding domain is connected via an extracellular spacer to a transmembrane domain, followed by cytoplasmic signaling domains such as the CD3ζ chain and co-stimulatory domains such as CD28 and 4-1BB. In August 2017, Kymriah^TM^ (tisa-cel) became the first genetically modified cell therapy for cancer to receive FDA approval. Tisa-cel is an autologous CAR-T cell product targeting CD19, a pan–B-cell marker present on all mature B cells as well as the majority of malignant B cells. In its registration trial for the treatment of pediatric and young adult patients with relapsed or refractory B-cell acute lymphocytic leukemia (B-ALL), Kymriah^TM^ achieved 82% (65/79) overall remission rate and a 66% probability of relapse-free survival at 18 months [58]. Since 2017, several additional autologous CAR-T cell therapies targeting CD19 or BCMA have been approved for the treatment of B-cell leukemia, lymphoma, and multiple myeloma (Table 2) [59–68]. As the technology matures, CD19 CAR-T cell therapy has advanced from third-line to second-line treatment for non-Hodgkin lymphoma, and clinical trials are already underway to evaluate CD19 CAR-T cell therapy as first-line treatment for high-risk large B-cell lymphoma [69]. In addition, CD19 CAR-NK cells have also demonstrated clinical promise [70], elevating NK cells as another potent effector cell type for immunotherapy.

**Table 2:** List of FDA-approved CAR-T cell therapies.

Trade Name	Cell Product	Target Antigen	Indication	References
Kymriah	tisagenlecleucel	CD19	Pediatric/young adult B-ALL, FL, DLBCL	[59–61]
Yescarta	axicabtagene ciloleucel	CD19	LBCL (DLBCL, HGBCL, PMBCL, tFL), FL	[62, 63]
Tecartus	brexucabtagene autoleucel	CD19	MCL, adult B-ALL	[64, 65]
Breyanzi	lisocabtagene maraleucel	CD19	LBCL (DLBCL, HGBCL, PMBCL, FL grade 3B)	[66]
Abecma	idecabtagene vicleucel	BCMA	Multiple myeloma	[67]
Carvykti	ciltacabtagene autoleucel	BCMA	Multiple myeloma	[68]

The success of CD19- and BCMA-targeted therapies highlight not only the promise but also the challenges associated with cell-based immunotherapy. Specifically, despite clinical trials evaluating CAR-T cells targeting dozens of antigens, only CD19 and BCMA CAR-T cells have received FDA approval to date, and no cell-based therapy has yet demonstrated comparable efficacy against solid tumors. To expand the applicability of cell-based therapy to a broad range of malignancies, engineering efforts now focus on not only optimizing the CAR protein, but also fine-tuning immune-cell biology to maximize anti-tumor efficacy.

### Genetic modifications to enhance T-cell potency

To achieve safe, potent, and durable therapeutic benefit, cell-based therapies must meet a number of performance criteria: (i) precise targeting of tumor cells and simultaneous avoidance of essential healthy tissue, (ii) complete coverage of the tumor population based on antigen recognition, and (iii) robust expansion and functional persistence of the effector cell population to eradicate existing tumor burden and provide long-term surveillance against tumor relapse. The first two objectives are most typically achieved through careful antigen selection and receptor engineering, a topic that has been extensively covered in several reviews[71, 72]. Here, we discuss strategies aimed at promoting the expansion and functional persistence of engineered cells post-infusion.

Different immune cell types are driven by fundamentally divergent biology that necessitates different strategies to optimize for therapeutic applications. T cells, as the dominant chassis to date, has been the focus of most engineering efforts in this area. T cells are a uniquely interesting substrate for engineering due to their natural diversity in phenotype and function. For example, effector T cells can execute potent cytotoxicity and explosive growth, but are relatively short-lived. In contrast, memory T cells are more tempered in effector functions, but can provide long-term persistence and in vivo surveillance. Importantly, despite efforts to neatly categorize T cells into distinct subtypes, advancements in epigenomic, transcriptomic, and proteomic analyses continue to reveal subtle gradations in T-cell biology and function [73]. For example, exhausted T cells, long described as a dysfunctional subset, are now understood to play a critical role in the effectiveness of immune checkpoint blockade therapy [74]. This increasingly complex picture of T-cell phenotype presents both challenges and opportunities for engineering, as it may be possible to genetically tune T-cell behavior to obtain an optimal combination of functions conducive to cancer therapy.

Early efforts in optimizing CAR-T cells’ anti-tumor efficacy focused on introducing co-stimulatory signaling into the CAR molecule, thus promoting T-cell proliferation and the intensity of effector outputs such as cytokine production and cytotoxicity upon CAR signaling [75]. With accumulating clinical experience, a number of genetic targets were identified as potential targets for ablation to improve T-cell function. For example, the success of anti–PD-1 checkpoint blockade led to the first US-based clinical trial involving CRISPR-edited cell therapy, in which T cells expressing an anti–NY-ESO-1 TCR were also edited by CRISPR/Cas9 to knock out PD-1 [76]. As another example, a patient with chronic lymphocytic leukemia responded to anti-CD19 CAR-T cell therapy only upon receiving a second dose of CAR-T cells, and the anti-tumor efficacy was attributed in large part to clonal expansion of a particular CAR-T cell that lacked any functional copy of TET2 (the patient had a congenital mutation in one copy of TET2, and the CAR transgene was randomly inserted into the other copy, thereby generating a double-knockout) [77]. This subsequently led to the intentional knockout of TET2 as a means to promote in vivo T-cell expansion and persistence [78]. More recently, the advent of CRISPR-based genetic libraries has enabled large-scale screening efforts to identify target genes whose knock-in or knock-out can enhance T-cell expansion, persistence, and anti-tumor efficacy. For example, the SWI/SNF family complex member ARID1A and the RAS GTPase-activating protein RASA2 have been identified through library screens and subsequently individually validated as knockout candidates to minimize T-cell exhaustion and improve T-cell function [13, 79]. Finally, numerous strategies are now under evaluation to “armor” tumor-targeting immune cells with chemokines, cytokines, and genetic circuitry designed to enhance anti-tumor efficacy, particularly against solid tumors that are otherwise protected by highly immunosuppressive tumor microenvironments [80, 81].

### Manufacturing challenges in cell-based immunotherapy

As additional gene targets are identified, technical challenges surrounding cell manipulation are rising to the forefront of the clinical translation process. To date, the vast majority of CAR-expressing immune cells, regardless of cell type, undergo lenti- or retroviral transduction to introduce the CAR-encoding transgene. However, the payload limitations of viral vectors quickly become a bottleneck when additional genetic elements need to be knocked in or knocked out of the cell product. Furthermore, the manufacturing of clinical-grade viral vectors is a major roadblock due to the high cost and limited availability of manufacturing slots. To overcome this chokepoint in therapy development, active research focuses on developing non-viral methods of gene delivery, including transposon-mediated gene integration [82], CRISPR-mediated homologous recombination [83], nanoparticle-mediated in situ gene transfer [84], among others.

The personalized nature of autologous therapy presents a significant manufacturing challenge, as a new product must be generated for each individual patient. Furthermore, most patients have already experienced multiple rounds of prior therapy with the potential to damage their immune cells, rendering the cell manufacturing process even more precarious. Allogeneic therapy using donor-derived cells present an attractive alternative to on-demand generation of patient-specific cell products, as the starting cell population from healthy donors could have substantially higher fitness levels compared to patient cells. In principle, the ability to pre-manufacture the cell product would also enable more extensive quality-control testing, potentially more complex genetic engineering, and the generation of sufficient doses for multiple patients from a single manufacturing campaign. To date, experimental allogeneic cell therapies have included αβ T cells with endogenous TCRs knocked out to minimize graft-versus-host, γδ T cells, NK cells, and invariant natural killer T (iNKT) cells; however, emerging data on allogeneic cell therapy indicate that durability of response remains a key challenge [85, 86].

## Successes and challenges in gene therapy—adeno-associated virus (AAV) therapies

Viral-based gene delivery systems rely on the natural ability of viruses to infect cells and deliver their genetic cargo. Recombinant Adeno-Associated Viral vectors (*AAVs*) are the preferred delivery vehicle for a number of gene therapies, due to their small size, high efficiency, low immunogenicity, and tunable tissue tropism. Wild type (WT) AAV is a non-enveloped parvovirus comprised of a single strand of DNA with *Rep* and *Cap* genes. Rep encodes four proteins that control viral replication, packaging, and genomic integration, and *Cap* encodes three subunits called VP1, VP2, and VP3, which make up the capsid coat in ratios of 1:1:10. The WT AAV genome is flanked by inverted terminal repeats (ITRs), which are critical for the replication and packaging of the DNA cargo within the capsid coat. AAV is so-named because it was initially identified as a contaminant in adenoviral preparations, and is not associated with any known disease. Typically, AAV-based gene therapy involves delivering a functional copy of a gene to replace a non-functional version; however, any nucleic acid can theoretically be delivered by AAV. AAV’s flexibility for different gene therapy applications is being increasingly recognized and adapted to deliver other therapeutic molecules, such as regulatory RNAs and gene editing platforms. Currently, the majority of gene-therapy products and products in late-stage clinical development use a gene replacement approach (Table 3). There are three approved AAV gene therapies in the United States (Luxturna^TM^, Zolgensma^TM^, and Hemgenix^TM^), and one with conditional approval in Europe (Rocktavian^TM^), but many more are being developed and trials are ongoing. While AAV-based approaches have been curative for some diseases, safety, efficacy, and manufacturing challenges remain.

**Table 3:** List of FDA- and EMA-approved AAV-based therapies.

Trade name	Proper name	AAV serotype	Target tissue(s)	Indication	Gene
Luxturna	voretigene neparvovec	AAV2	eye	Leber congenital amaurosis	*RPE65*
Zolgensma	onasemnogene abeparvovec	AAV9	Brain, muscle	Spinal muscular atrophy	*SMN*
Rocktavian	valoctocogene roxaparvovec	AAV5	liver	Hemophilia A	*F8*
Hemgenix^TM^	etranacogene dezaparvovec	AAV5	liver	Hemophilia B	*F9*

### Basics of vector design for AAV based gene therapy

#### Design of the AAV cargo

In order to use an AAV for gene therapy applications, the AAV genome (*Rep* and *Cap* gene) is removed, and replaced by a therapeutic expression cassette. This cassette consists of a regulatory element (promoter), the cDNA encoding the functional gene, and a poly A signal. The cassette is flanked by the only two remaining viral genome elements, the two 140 bp ITRs. The total size of the expression cassette including the ITRs should be ~4.8 kb, although larger inserts at ~5 kb have been successfully packaged at a cost of reduced efficiency. By using a tissue-specific promoter, transgene expression is limited to the target tissue(s), preventing expression in antigen-presenting cells, which can trigger transgene-specific immune responses. The therapeutic transgene can be codon optimized to improve its translation; however, this modification often increases the number of unmethylated CpGs, which can contribute to enhanced immune responses to the transgene [87, 88], so codon optimization should be carried out without increasing the CpG content.

#### Design of the AAV capsid

The AAV capsid determines the tissue specificity or “tropism” for each AAV serotype. Hundreds of AAV serotypes of human or primate origin have now been described. While the mechanisms are not completely elucidated, most AAV serotypes use a co-receptor (often a membrane protein) and receptor (a glycan) to gain entry to cells. Upon receptor and co-receptor binding, AAV is endocytosed, escapes the endosome, travels to the nucleus and is uncoated. In the nucleus, the single-stranded DNA is converted to double-stranded DNA, circularized, and subsequently remains as an episome. The capsid proteins are degraded in the proteasome and presented on major histocompatibility complexes. New AAV serotypes are being identified through capsid engineering to create serotypes with enhanced specificity to tissues of interest [89]. AAV2 has the widest tissue tropism of all known serotypes, likely because it is able to utilize numerous receptors and co-receptors. The choice of AAV capsid for gene therapy applications should target disease-relevant tissues with as much specificity as possible.

#### AAV packaging

For gene therapy applications, AAV packaging is accomplished in human (HEK293) or insect (sf9) cells, which can be adherent or grown in suspension. Use of these two cell types results in differences in the types of post-translational modifications that are present after packaging, as well as the integrity of the packaged DNA [90, 91]. Because the genes involved in replication and packaging are removed from the expression cassette, the Rep and Cap genes need to be supplied in trans along with an adenoviral helper plasmid to accomplish AAV packaging. Thus, three plasmids are triple transfected to cells in order to package AAV. Following packaging, a variety of purification methods can be used, including cesium chloride centrifugation [92], ion exchange chromatography [93], and affinity purification [94]. It is common for contract development and manufacturing organizations (CDMOs) to handle this task for GMP-grade vector, although many companies are developing their own manufacturing capabilities.

### Successes

*Luxturna*^TM^ for Leber congenital amaurosis (LCA): In 2017, the first AAV-based gene therapy was FDA-approved for the treatment of a form of hereditary blindness called LCA. LCA is comprised of 23 different genetically defined retinal disorders that lead to vision loss [95]. The drug Luxturna is now approved for LCA patients harboring mutations in both alleles of the *RPE65* gene [96]. Mutations affect the production or function of RPE65, which is expressed in retinal epithelial cells. The *RPE65* gene encodes a retinoid isomerohydrolase that is needed to produce a chromophore for phototransduction, called 11-cis retinal [97]. Photoreceptors lacking RPE65 will degenerate and lead to vision loss. Individuals with homozygous mutations experience progressive vision loss during the first year of life. Luxturna uses AAV2 to carry the human *RPE65* gene under the control of the beta actin promoter, leading to dramatic restoration of vision to those who receive this therapy.

*Zolgensma*^TM^ for Spinal Muscular Atrophy type I: The second AAV-based gene therapy approved in the United States is Zolgensma^TM^, which received FDA approval in May 2019 for children under the age of 2 with infantile-onset spinal muscular atrophy (SMA) type I (AKA Werdnig-Hoffman disease). Zolgensma^TM^ is indicated for babies with homozygous loss of function mutations in the *SMN1* gene. SMN is a protein that is necessary for development of alpha motor neurons. In the absence of SMN protein, the spinal cord and brainstem degenerate, leading to weakness in the limbs, trunk, swallowing and breathing muscles. Children born with SMA type I are not able to sit unassisted, have difficulty breathing and swallowing, and usually die within the first year of life. Zolgensma^TM^ uses AAV9 to deliver a copy of the *SMN1* gene, driven by a CMV promoter. Because SMN expression is needed in both neurons and muscle cells, the therapy utilizes a promoter with broad tissue expression. This therapy appears to be most efficacious if administered prior to 6 months of age because post-natal delivery of *SMN1* stabilizes, but does not reverse the disease process. However, treatment with Zolgensma^TM^ has been life-changing for children born with SMA type I, with many able to breathe, eat and even walk on their own. Reports five years post-dosing suggest that the durability of Zolgensma^TM^ is high. This therapy was the first systemically delivered AAV-based therapy and as such, has paved the way for many more that require intravenous administration and systemic delivery.

*Roctavian*^TM^ for Hemophilia A and Hemgenix^TM^ for Hemophilia B: Hemophilia is a rare inherited blood clotting disorder caused by deficiency of factor VIII (hemophilia A) or factor IX (hemophilia B). Patients experience episodes of excessive bleeding affecting soft tissues and joints. Gene therapy programs to restore these factors are being actively pursued in both disease categories; however the first commercially successful program was achieved in hemophilia A [98]. Roctavian^TM^ has EMA conditional approval for treatment of hemophilia A, which uses AAV5 to deliver a FVIII cDNA under the regulation of a liver specific promoter. The therapy restores FVIII at high doses, resulting in reduced need for externally provided FVIII (99% reduction). However, in the phase III study, loss of transgene is apparent with time, from an average of 64% of FVIII activity at 1 year down to 24% by year 4 (J.P. Morgan presentation 11 Jan 2021). The reason for the loss of transgene is not clear, but may relate to the high immunogenicity of FVIII or natural turnover of hepatocytes with time. Shortly after Rocktavian’s conditional EMA approval in 2022, for hemophilia A, Hemgenix^TM^ received FDA approval for hemophilia B. Like Roctavian^TM^, Hemgenix^TM^. Uses the AAV5 vector to deliver FIX under the control of a liver promoter.

### Challenges

The immune response to AAV poses the greatest challenge for successful AAV-based therapies. Three components of AAV vectors can trigger immunity: 1) AAV-capsid, 2) unmethylated CpGs in the nucleic acid cargo and 3) the protein transgene. Immune responses prevent vector re-administration, thus limiting AAV to a single dose. Furthermore, because humans are naturally exposed to wild type AAV (wtAAV), an estimated 30–70 % of individuals have pre-existing immunity by the time they reach adulthood [99, 100]. Therefore, successful AAV-based therapies require that AAV vectors overcome preexisting immune responses in patients. Strategies to dose in the presence of pre-existing immunity have thus far been unsuccessful, but researchers are testing whether plasmapheresis [101], capsid decoys [102], or the use of enzymes to cleave circulating IgG will have utility [103].

#### Safety and toxicity

Although clinical trials with AAV are still in their early stages, AAV is generally considered safe when dosed in vivo. Most AAV serotypes are sequestered in the liver and as such the most common adverse event is liver toxicity, clinically observed as an elevation of liver enzymes [104–107]. In hemophilia gene therapy trials, patients experienced liver toxicity that could be resolved with a short course of prednisone treatment [108]. These SAEs were attributed to CD8 T cell mediated attack against hepatocytes presenting capsid on MHC [106, 109]. Steroid treatment could be discontinued once the viral capsid was shed [110]. Other adverse events include hepatic hepatocellular carcinoma, which was observed in a single patient who also had several co-morbidities, and this event led to a pause in a clinical trial for hemophilia B.

The most prevalent adverse events (AEs) in high-dose AAV administration are thrombotic microangiopathy (TMA) or atypical hemolytic uremic syndrome (aHUS), both of which are likely due to complement recognition of the AAV capsid. TMA has been observed in several spinal muscular atrophy and Duchenne muscular dystrophy (DMD) subjects treated with high-dose, systemically delivered AAV vectors. Based on reports from Pfizer, Solid Biosciences, and Novartis [111, 112], there is ample evidence that this adverse event likely relies on the presence of anti-AAV antibodies [113]. TMA has led to clinical holds for two different DMD trials, but symptoms were resolved using eculizumab, a C5 complement inhibitor, for most patients, although two DMD patients died. Trials for X-linked microtubular myopathy, in which AAV8 was used to deliver the *MTM1* gene, have resulted in patient deaths from liver failure, which may be a component of the disease. Taken together, low-dose AAV treatment is supported by a strong safety profile, especially in the presence of steroids, but higher AAV doses appear to trigger TMA or aHUS in a subset of patients with pre-existing antibodies or rapid antibody responses. Dosing protocols are increasingly adding immunosuppression to avoid these AEs. It is important to note that the methods for vector production and titration are not standardized and so dosages, empty to full capsid ratios and impurities (such as endotoxins) could differ across trials and may be a contributing factor to some of these toxicities.

#### Durability

While AAV-based approaches have been considered to be “one and done”, all available evidence suggests that re-dosing of the therapeutic will be needed to achieve a long-lasting therapy. The durability of the transgene can be compromised by the lifespan of the target cell or the immune response to the transgene, but for each target tissue and vector, these risks will need to be established independently. Since an adaptive immune response develops after initial dosing, new doses of vector will be neutralized before reaching its target tissue. Changing AAV capsids does not seem to be a valid solution for avoiding immunity, because of the high capacity for cross-reactivity. Therefore AAV-based approaches are limited to a single dose unless additional measures are taken to reduce antibodies and T cells from prior exposure. Re-dosing can be achieved if the initial dose is provided in the presence of strong immunosuppression, but these studies are still in their early stages [114, 115]. Transgene immunity can be a major challenge for treating patients with null mutations due to lack of tolerization of the transgene product, which has been observed in hemophilia A [116, 117] and DMD (Bonneman, American Society for Gene and Cell Therapy, 2022 Annual meeting). Several patients developed anti-transgene responses manifesting as myositis in three different DMD gene therapy trials, and it has been suggested that the target is the transgene. This observation led several companies to change their inclusion criteria, only including patients whose endogenous gene contains the same elements that are contained within the transgene. Therefore, while initial results have been extremely promising, the issue of durability will likely need to be addressed.

### Manufacturing, scale up, and costs

The production, scale-up, and costs of AAV-based therapies are major obstacles to their widespread success. This issue is particularly problematic for gene therapy applications requiring high vector doses, such as those targeting skeletal muscle [118]. There are a number of steps involved in creating the final biological product, including the source and type of cells for packaging, the manufacturing method for plasmids used for transfection, the choice of helper plasmid, the stability and purity of the final product, and determining titers and empty-to-full ratios, among others [119]. The manufacturing process and how it impacts these characteristics are largely unknown; however with enhanced transparency, the impact of manufacturing on clinical efficacy will be made clearer. Manufacturing capabilities are expanding in the US and in Europe, and it is hoped that AAV vector production will be able to meet demand as trials and commercialization progress. Currently, costs are astronomical, but as process development becomes more streamlined, it is anticipated that costs should begin to decline.

## Data sharing statement

Data sharing not applicable to this article as no datasets were generated or analyzed during the current study.
